# Defining disrespect and abuse of women in childbirth: a research, policy and rights agenda

**DOI:** 10.2471/BLT.14.137869

**Published:** 2014-10-06

**Authors:** Lynn P Freedman, Kate Ramsey, Timothy Abuya, Ben Bellows, Charity Ndwiga, Charlotte E Warren, Stephanie Kujawski, Wema Moyo, Margaret E Kruk, Godfrey Mbaruku

**Affiliations:** a Mailman School of Public Health, Columbia University, 60 Haven Avenue (B3), New York, NY 10032, United States of America.; b Population Council, Nairobi, Kenya.; c Ifakara Health Institute, Dar es Salaam, United Republic of Tanzania.

In the field of maternal and newborn health, there have been calls to prioritize the intra-partum period and promote facility delivery to meet maternal and newborn mortality reduction goals. This aim is based on a decade of epidemiological work identifying causes of death, systematically reviewing effective interventions, and modelling the impact of intervention coverage on mortality.^1^ Yet increases in facility delivery and in known effective interventions provided in those facilities have not always had the expected impact.^2^ This has led to growing concern about the quality of the care that women are experiencing during labour and delivery.

International law holds that the right to health requires health services that are available, accessible, acceptable and of good quality. But despite numerous official interpretations and guidance documents applying this right to childbirth,^3^ reports of disrespectful and abusive treatment during labour and delivery continue to appear in many parts of the world.

Together, clinical guidelines and human rights law create a set of normative standards that form a vision for a health system that is people-centred, responsive and effective. The challenge is to implement such a system equitably and sustainably. Health systems are deeply embedded in society’s broader social and political dynamics, which can contribute to disrespect and abuse of women giving birth. A strategy to address this situation needs to take local drivers of disrespect and abuse seriously, using both top-down and bottom-up approaches to incorporate normative standards into routine practice.

Evidence on the nature and frequency of disrespect and abuse is essential for effective programmes, policy and advocacy. Yet, in the existing literature, there is no definition of disrespect and abuse that can be used to study its prevalence or evaluate interventions to address it. Formal legal definitions do not resolve this definitional problem.

Here we report on the approach to defining disrespect and abuse developed by two affiliated projects (which are part of a broader global effort) seeking to promote respectful maternal care in Kenya and the United Republic of Tanzania. These projects combine epidemiological research on prevalence, implementation research on interventions, and advocacy efforts to create policy change. They are the first initiatives, to our knowledge, to systematically measure the prevalence and nature of disrespect and abuse.^4^

## From description to definition

Most of the literature on disrespect and abuse is anecdotal, or consists of case studies of specific incidents or sites. The reported forms of disrespect and abuse have been usefully grouped into seven categories: physical abuse, non-consented care, non-confidential care, non-dignified care, discrimination based on patient attributes, abandonment of care and detention in facilities.^5^ These categories describe types of disrespect and abuse that happen in health facilities, but do not define it in terms of the characteristics of health-care provider behaviour, facility conditions or other factors that could be construed as disrespectful and abusive.

We set out to create a robust definition that would capture both individual disrespect and abuse (i.e. specific provider behaviours experienced or intended as disrespectful or humiliating, such as slapping or scolding of women) and structural disrespect and abuse (i.e. systemic deficiencies that create a disrespectful or abusive environment, such as an overcrowded and understaffed maternity ward where women deliver on the floor, alone, in unhygienic conditions).

Such a definition could be used by researchers measuring prevalence and studying interventions; health-system managers seeking to transform their facilities; professional associations trying to shift the values and norms of their members; and advocates and activists mobilizing for accountability and change.

## Definition building blocks

The broadest definition of disrespect and abuse is set by the right to health. To exercise their right to available, accessible, acceptable and good quality care, pregnant women need access to the infrastructure, equipment and staff required for routine and emergency obstetric and newborn care. National policies typically supply detailed standards in each of these areas.

However, defining disrespect and abuse solely as a deviation from the right to health presents a dilemma. If every delivery in a facility with infrastructure, staff and equipment that do not meet global or national policy standards is defined as being disrespectful and abusive, then prevalence could be 100%. This is clearly not a useful way to establish the baseline for interventions. Yet neither of our country teams wanted to ignore the human rights standard or imply that their citizens are entitled to less.

Conversely, a definition of disrespect and abuse based on the actual experience of violations from the perspectives of both victim and perpetrator will be limited, especially when aspects of disrespect and abuse are so common among providers or so expected by patients as to be normalized in the health system. However, building a definition from the experiential level starts a process that engages key stakeholders (patients, families, providers and administrators). Listed below are the experiential building blocks we developed to define disrespect and abuse.

### Behaviour that, by local consensus, constitutes disrespect and abuse

Women’s experiences of disrespect and abuse depend less on normative standards than on the unwritten norms in their locality. A specific set of behaviours or conditions will be agreed by all stakeholders to constitute disrespect and abuse. This consensus list forms the core of our definition.

### Subjective experience

If a woman experiences treatment as disrespectful or abusive, even if it is not included in the list above, does it constitute disrespect and abuse? What if a woman experiences conditions or behaviours in this way, but the providers, often deeply distressed themselves by their work environment, are actually doing their best? If our goal is to protect women’s rights and dignity in childbirth, and to increase facility delivery, then it matters if a woman (or her accompanying family members) experiences her treatment as disrespectful and abusive. Such an experience is likely to influence future decisions about where to deliver and whether to recommend that facility to others,^6^ and valuing patient experience is the essence of patient-centred health systems.

### Intentionality

What if the woman does not experience an action as disrespectful or abusive, but the provider intends it as such? Our teams agreed that the definition should include actions that the provider intends to be harmful, but that such intent should not be a requirement of disrespect and abuse (i.e. unintended disrespect and abuse should also be included).

**Fig. 1:**
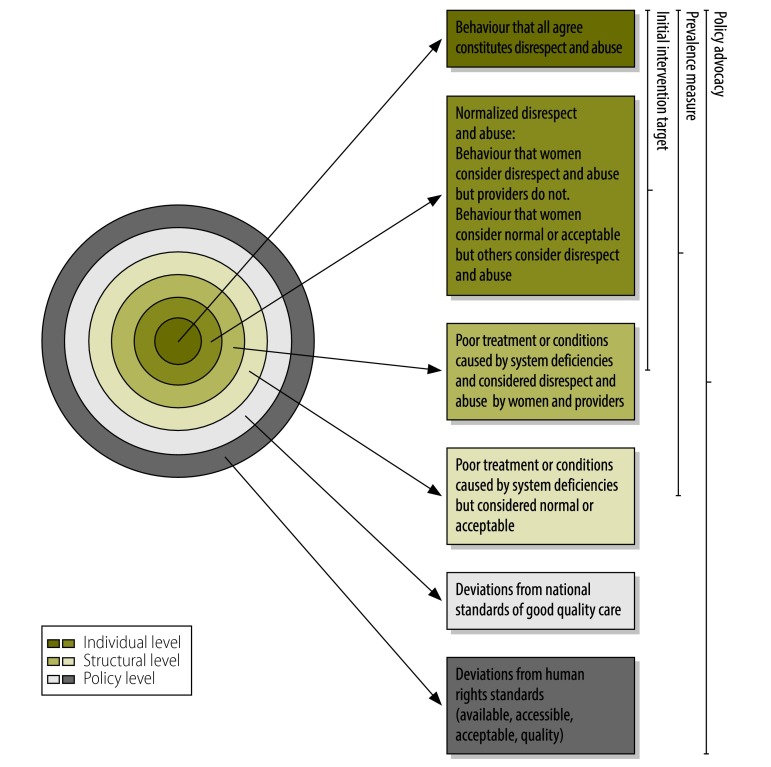
Defining disrespect and abuse of women in childbirth

To be useful in practice, the definition of disrespect and abuse requires both normative standards and experiential building blocks. To combine these different approaches, we drew a set of circles (Fig. 1). As normalized behaviour is challenged and changed, leading to a reduction in disrespect and abuse, the diameter of the innermost circle should expand in relation to the others (Fig. 1). Using this diagram, our teams were able to make strategic decisions about using different definitions of disrespect and abuse for different purposes.

This diagram has proven to be an effective tool for initiating discussion of disrespect and abuse at local, national and global levels. When community representatives, providers and administrators meet to discuss their different perspectives on what constitutes and drives disrespect and abuse, the diagram gives each experience an acknowledged place in the discussion. When different methods for measuring disrespect and abuse – such as multiple approaches to self-report as well as third-party observation – yield dramatically divergent prevalence estimates (as they did in both our projects), the dynamic diagram helps researchers to make sense of findings and to shape a principled but pragmatic response.

## Conclusion

The growing global movement to promote respectful maternal care has begun to make strategic use of normative standards defined in law and policy. But our projects recognized that simply promoting abstract standards through advocacy and education – or even through legal enforcement and punishment – is unlikely to solve the problem of disrespect and abuse. The abstract standards could only acquire meaning over time by careful attention to the lived experience of disrespect and abuse, and to the deeper dynamics of power that underlie it.

As a starting point for research and action, we define disrespect and abuse in childbirth as interactions or facility conditions that local consensus deems to be humiliating or undignified, and those interactions or conditions that are experienced as or intended to be humiliating or undignified. Over time, we expect this definition to converge with both national and human rights standards for good quality and respectful maternal care. By combining the experiential building blocks and the normative standards, this definition provides a platform to bring divergent groups together to challenge unacceptable social norms and poor health-system practices. Although research is underway in the two projects to measure prevalence and test interventions, more is required to understand the drivers and consequences of disrespect and abuse in these and other settings globally. Development of interventions to reduce disrespect and abuse, with clearly articulated theories of change and appropriate strategies to assess implementation, will be critical to building an effective global movement for respectful maternal care.
